# Vacation Days Taken, Work During Vacation, and Burnout Among US Physicians

**DOI:** 10.1001/jamanetworkopen.2023.51635

**Published:** 2024-01-12

**Authors:** Christine A. Sinsky, Mickey T. Trockel, Lotte N. Dyrbye, Hanhan Wang, Lindsey E. Carlasare, Colin P. West, Tait D. Shanafelt

**Affiliations:** 1 American Medical Association, Chicago, Illinois; 2 Stanford University School of Medicine, Palo Alto, California; 3 School of Medicine, University of Colorado Anschutz Medical Campus, Aurora; 4 Division of General Internal Medicine, Mayo Clinic, Rochester, Minnesota; 5 Division of Biomedical Statistics and Informatics, Mayo Clinic, Rochester, Minnesota

## Abstract

**Importance:**

Vacation has been shown to be an important restorative activity in the general population; less is known about physicians’ vacation behaviors and their association with burnout and professional fulfillment.

**Objective:**

To examine the number of vacation days taken per year and the magnitude of physician work while on vacation and their association with physician burnout and professional fulfillment, by individual and organizational characteristics.

**Design, Setting, and Participants:**

This cross-sectional survey of US physicians was conducted between November 20, 2020, and March 23, 2021. Data analysis was performed from March to July 2023.

**Main Outcomes and Measures:**

Burnout was measured using the Maslach Burnout Index, and professional fulfillment was measured using the Stanford Professional Fulfillment Index. Number of vacation days taken in the last year, time spent working on patient care and other professional tasks per typical vacation day (ie, work on vacation), electronic health record (EHR) inbox coverage while on vacation, barriers to taking vacation, and standard demographics were collected.

**Results:**

Among 3024 respondents, 1790 of 3004 (59.6%), took 15 or fewer days of vacation in the last year, with 597 of 3004 (19.9%) taking 5 or fewer days. The majority, 2104 respondents (70.4%), performed patient care–related tasks on vacation, with 988 of 2988 (33.1%) working 30 minutes or more on a typical vacation day. Less than one-half of physicians (1468 of 2991 physicians [49.1%]) reported having full EHR inbox coverage while on vacation. On multivariable analysis adjusting for personal and professional factors, concern about finding someone to cover clinical responsibilities (odds ratio [OR], 0.48 [95% CI, 0.35-0.65] for quite a bit; OR, 0.30 [95% CI, 0.21-0.43] for very much) and financial concerns (OR, 0.49 [95% CI, 0.36-0.66] for quite a bit; OR, 0.38 [95% CI, 0.27-0.54] for very much) were associated with decreased likelihood of taking more than 3 weeks of vacation per year. Taking more than 3 weeks of vacation per year (OR, 0.66 [95% CI, 0.45-0.98] for 16-20 days; OR, 0.59 [95% CI, 0.40-0.86] for >20 days vs none) and having full EHR inbox coverage while on vacation (OR, 0.74; 95% CI, 0.63-0.88) were associated with lower rates of burnout on multivariable analysis, whereas spending 30 minutes or longer per vacation day on patient-related work (OR, 1.58; 95% CI, 1.22-2.04 for 30-60 minutes; OR, 1.97; 95% CI, 1.41-2.77 for 60-90 minutes; OR, 1.92; 95% CI, 1.36-2.73 for >90 minutes) was associated with higher rates of burnout.

**Conclusions and Relevance:**

In this cross-sectional study of 3024 physicians, the number of vacation days taken and performing patient-related work while on vacation were associated with physician burnout. System-level efforts to ensure physicians take adequate vacation and have coverage for clinical responsibilities, including EHR inbox, may reduce physician burnout.

## Key Points

**Question:**

Are vacation days taken and working while on vacation associated with physician burnout?

**Findings:**

In this cross-sectional study of 3024 US physicians, 59.6% took 3 weeks of vacation or less per year, and 70.4% worked while on vacation on a typical vacation day; both findings were associated with higher rates of burnout. Full electronic health record inbox coverage was associated with lower rates of working while on vacation and with lower burnout.

**Meaning:**

These findings suggest that support for taking vacation and efforts to reduce physicians’ obligations to perform patient care–related tasks while on vacation, such as providing full electronic health record inbox coverage, should be considered to prevent physician burnout.

## Teaser

This cross-sectional study examines the number of vacation days taken per year and the magnitude of physician work while on vacation and their association with physician burnout and professional fulfillment.

## Introduction

Vacation has been shown to be an important restorative activity in the general population. Time away from work provides an opportunity to rest and recharge and is associated with benefits to both employer and employee, including improved job performance, greater productivity, enhanced creativity, greater job satisfaction, enhanced attention to personal relationships, lower absenteeism, less turnover, and lower rates of burnout.^1,2,3,4,5,6,7,8^ Taking vacation is also associated with improved physical and mental health, including lower risk of cardiovascular mortality, reduced cellular-level markers of stress, and fewer symptoms of depression and anxiety.^9,10,11,12,13,14^ Despite these benefits, less than one-half (48%) of US workers with paid time off use their full allotted time.^15^ Fully detaching from work while on vacation is also important and has been shown to improve productivity and reduce emotional exhaustion,^1^ whereas working during vacation negatively influences health and well-being after vacation.^8^ Yet, fully detaching from work during time off is not universal. Among workers with a postgraduate degree, 41% report often or extremely often responding to email or other messages outside of their normal work hours.^15^

Less is known about the vacation behaviors of US physicians and the institutional policies and practices that facilitate or hinder taking vacation. A 2023 survey^16^ of a convenience sample composed of 9175 physicians in 29 specialties found that 1 in 3 physicians took 2 weeks or less of vacation each year. A 2020 study^17^ of 490 physicians found that being able to disconnect from work is associated with lower emotional exhaustion and depersonalization, the 2 key dimensions of burnout. Although variation in physician vacation behavior by gender is unknown, female physicians report worse work-life integration,^18,19^ spending more time on the electronic health record (EHR), including after hours,^20,21,22,23,24^ and higher and more rapidly rising rates of burnout^25^ than male colleagues. The present study aims to characterize vacation behavior in a nationally representative sample of US physicians, to identify personal and professional characteristics associated with time spent on patient care–related work while on vacation and the number of vacation days taken, and to analyze the association of vacation characteristics with rates of burnout and professional fulfillment.

## Methods

As previously reported,^26^ between November 20, 2020, and March 23, 2021, we surveyed a representative sample of physicians of all specialties listed in the American Medical Association Physician Professional Data using a methodological approach similar to the 2011,^27^ 2014,^28^ and 2017^29^ surveys. The Physician Professional Data, previously named the American Medical Association Masterfile, is a nearly complete record of all US physicians. Among the 3671 physicians who received an invitation to participate in the mailed survey, 1162 (31.7%) completed the survey. Of the 90 000 physicians invited to participate in the electronic survey, 6348 (7.1%) completed the survey.^26^ Participation was voluntary and all responses were anonymous. Informed consent was implied upon completing the survey. A random subset of participants received a subsurvey with items about vacation. As previously reported,^26^ detailed analysis comparing the demographic characteristics of participating physicians with all 897 107 practicing US physicians, as well as a secondary survey of nonresponders, suggested that participants were representative of US physicians (see the eAppendix in Supplement 1). The Stanford and Mayo Clinic institutional review boards approved this cross-sectional study, which followed the Strengthening the Reporting of Observational Studies in Epidemiology (STROBE) reporting guidelines.

The vacation subsurvey included items about vacation days taken per year, inbox coverage while on vacation, time spent working on patient care and other professional tasks per typical vacation day (ie, work on vacation [WOV]), and barriers to taking vacation (eAppendix in Supplement 1). Physicians were asked, “Using the definition of vacation that applies to your practice, how many days of vacation did you take in the last 12 months?” Response options were none, 1 to 5 work days (about 1 week), 6 to 10 work days, 11 to 15 work days, 16 to 20 work days, or more than 20 work days. Physicians were asked whether they had full EHR inbox coverage while on vacation, with response options of yes or no. Physicians were also asked, “On a typical vacation day in the last year, how much time did you spend responding to patient-related phone calls, inbox messages in the EHR and on other work-related email?” with response options of none, 1 to 30 minutes, 30 to 60 minutes, 60 to 90 minutes, or more than 90 minutes. Finally, physicians were asked to rate how much of a barrier to taking vacation each of the following dimensions was for them: finding someone to cover clinical responsibilities, financial impact on professional compensation, and the volume of EHR inbox work to be faced on return, with response options for each dimension of not at all, a little bit, somewhat, quite a bit, or very much.

The Maslach Burnout Inventory was used under license with Mind Garden, Inc, to assess the emotional exhaustion and depersonalization dimensions of burnout.^30^ The professional fulfillment subscale of the Stanford Professional Fulfillment Index was used to measure professional fulfillment with scores normalized on a scale of 0 to 10 (higher scores are more favorable) according to convention.^31^ Standard demographic information, including age, gender, relationship status, specialty, hours worked per week, and primary practice setting was collected. In addition, physicians were asked what percentage of their compensation was based on productivity, with response options of none, less than 25%, 25% to 50%, 51% to 75%, or greater than 75%.

### Statistical Analysis

Data analysis was performed from March to July 2023. Demographic and professional characteristics of physicians and their responses to questions on barriers to taking vacation were summarized using standard descriptive statistics. Associations between vacation items and demographic and professional factors, as well as associations between vacation items and overall burnout, were examined using χ^2^ tests. Emotional exhaustion, depersonalization, and professional fulfillment scores were compared using *t* tests and 1-way analysis of variance. Multivariable logistic regression was used to identify variables associated with overall burnout, professional fulfillment, and the 3 vacation items. For all analyses, statistical significance was set at 2-tailed *P* < .05. All statistical analyses were conducted using R statistical software version 4.1.2 (R Core Team).

## Results

Among the 3128 physicians invited to complete the vacation subsurvey, 3024 (96.7%) completed at least 1 vacation item; not all respondents completed all items, so denominators vary by item. Among respondents, 1790 of 3004 (59.6%) took 15 or fewer days (≤3 weeks) of vacation, 1193 of 3004 (39.7%) took 6 to 15 days (1-3 weeks) of vacation, and 597 of 3004 (19.9%) took 5 or fewer days of vacation in the previous 12 months. Less than one-half of physicians (1468 of 2991 physicians [49.1%]) reported full coverage for the EHR inbox while on vacation. On a typical vacation day, 2104 of 2988 physicians (70.4%) reported performing some WOV, with 1116 of 2104 (37.3%) working 1 to 30 minutes, 548 of 2104 (18.3%) working 30 to 60 minutes, 218 of 2104 (7.3%) working 60 to 90 minutes, and 222 of 2104 (7.4%) working more than 90 minutes. Results by demographics, specialty, primary practice setting, and percentage of compensation based on productivity are shown in Table 1.

**Table 1.** Physician Vacation and Patient-Related Tasks Performed on Vacation Across Demographic and Professional Factors of 3024 US Physicians

Variable	Participants, No. (%)							
	All (N = 3024)	Vacation days in last 12 mo			Time spent on patient-related work tasks during vacation, min/d^a^			Full EHR inbox coverage while on vacation
		≤5	6-15	>15	0	1-30	≥30	
All physicians	NA	597 (19.9)	1193 (39.7)	1214 (40.4)	884 (29.6)	1116 (37.3)	988 (33.1)	1468 (49.1)
Gender								
Male	1873 (62.0)	354 (19.0)	728 (39.1)	780 (41.9)	583 (31.5)	713 (38.5)	555 (30.0)	900 (48.5)
Female	1145 (37.9)	243 (21.4)	461 (40.6)	432 (38.0)	299 (26.4)	402 (35.5)	430 (38.0)	566 (50.1)
Other or missing	6^b^	NA	NA	NA	NA	NA	NA	NA
Age, y								
<35	121 (4.2)	30 (25.2)	48 (40.3)	41 (34.5)	52 (43.7)	39 (32.8)	28 (23.5)	65 (54.2)
35-44	748 (25.7)	155 (20.8)	299 (40.1)	291 (39.1)	198 (26.6)	294 (39.5)	252 (33.9)	358 (48.3)
45-54	715 (24.6)	131 (18.4)	298 (41.9)	283 (39.7)	164 (23.2)	276 (39.0)	267 (37.8)	361 (50.8)
55-64	790 (27.1)	145 (18.4)	335 (42.5)	308 (39.1)	215 (27.4)	285 (36.4)	284 (36.2)	373 (47.8)
≥65	536 (18.4)	115 (21.8)	170 (32.3)	242 (45.9)	214 (40.9)	181 (34.6)	128 (24.5)	258 (49.0)
Missing	114^b^	NA	NA	NA	NA	NA	NA	NA
Relationship status								
Single	318 (10.5)	93 (29.2)	125 (39.3)	100 (31.4)	105 (33.7)	104 (33.3)	103 (33.0)	146 (46.5)
Married	2525 (83.7)	471 (18.8)	994 (39.6)	1044 (41.6)	735 (29.4)	940 (37.6)	823 (32.9)	1237 (49.5)
Partnered	142 (4.7)	28 (19.9)	57 (40.4)	56 (39.7)	34 (24.3)	60 (42.9)	46 (32.9)	65 (46.4)
Widowed	31 (1.0)	4 (14.3)	13 (46.4)	11 (39.3)	8 (26.7)	10 (33.3)	12 (40.0)	16 (55.2)
Missing	8^b^	NA	NA	NA	NA	NA	NA	NA
Age of youngest child, y								
None	466 (15.5)	109 (23.5)	187 (40.4)	167 (36.1)	152 (33.0)	164 (35.6)	145 (31.5)	214 (46.3)
<5	415 (13.8)	91 (22.1)	173 (42.1)	147 (35.8)	117 (28.5)	167 (40.7)	126 (30.7)	210 (51.0)
5-12	543 (18.1)	95 (17.6)	208 (38.4)	238 (44.0)	133 (24.7)	198 (36.7)	208 (38.6)	273 (50.8)
13-18	407 (13.6)	84 (20.7)	171 (42.1)	151 (37.2)	105 (26.1)	143 (35.6)	154 (38.3)	200 (49.6)
19-22	309 (10.3)	41 (13.3)	136 (44.2)	131 (42.5)	77 (25.1)	117 (38.1)	113 (36.8)	149 (48.5)
>23	861 (28.7)	173 (20.3)	307 (35.9)	374 (43.8)	293 (34.6)	318 (37.6)	235 (27.8)	411 (48.5)
Missing	23^b^	NA	NA	NA	NA	NA	NA	NA
Specialty								
Anesthesiology	124 (4.1)	10 (8.1)	20 (16.3)	93 (75.6)	61 (49.2)	32 (25.8)	31 (25.0)	44 (36.1)
Dermatology	63 (2.1)	9 (14.5)	23 (37.1)	30 (48.4)	8 (12.7)	32 (50.8)	23 (36.5)	29 (46.0)
Emergency medicine	175 (5.8)	53 (30.8)	78 (45.3)	41 (23.8)	95 (54.6)	45 (25.9)	34 (19.5)	75 (43.6)
Family medicine	224 (7.5)	47 (21.0)	100 (44.6)	77 (34.4)	55 (24.7)	77 (34.5)	91 (40.8)	114 (51.1)
General surgery	103 (3.4)	22 (21.6)	39 (38.2)	41 (40.2)	26 (25.7)	49 (48.5)	26 (25.7)	44 (44.0)
Surgery subspecialty	239 (8.0)	47 (19.8)	107 (45.1)	83 (35.0)	40 (17.2)	94 (40.5)	98 (42.2)	99 (41.8)
General internal medicine	209 (7.0)	61 (29.6)	89 (43.2)	56 (27.2)	68 (32.7)	65 (31.2)	75 (36.1)	104 (50.2)
Internal medicine subspecialty	301 (10.0)	65 (21.7)	126 (42.0)	109 (36.3)	60 (20.5)	110 (37.5)	123 (42.0)	161 (54.0)
Neurology	91 (3.0)	16 (17.6)	38 (41.8)	37 (40.7)	15 (16.7)	36 (40.0)	39 (43.3)	43 (47.8)
Neurosurgery	31 (1.0)	4 (12.9)	15 (48.4)	12 (38.7)	6 (19.4)	13 (41.9)	12 (38.7)	12 (38.7)
Obstetrics and gynecology	123 (4.1)	22 (18.0)	41 (33.6)	59 (48.4)	38 (31.1)	39 (32.0)	45 (36.9)	68 (55.7)
Ophthalmology	126 (4.2)	27 (22.0)	49 (39.8)	47 (38.2)	44 (34.9)	50 (39.7)	32 (25.4)	66 (52.4)
Orthopedic surgery	136 (4.5)	23 (16.9)	51 (37.5)	62 (45.6)	28 (20.9)	58 (43.3)	48 (35.8)	65 (48.5)
Other	190 (6.3)	40 (21.2)	84 (44.4)	65 (34.4)	45 (23.9)	77 (41.0)	66 (35.1)	88 (47.3)
Otolaryngology	19 (0.6)	7 (36.8)	7 (36.8)	5 (26.3)	7 (36.8)	6 (31.6)	6 (31.6)	9 (47.4)
Pathology	80 (2.7)	8 (10.0)	27 (33.8)	45 (56.2)	27 (34.2)	33 (41.8)	19 (24.1)	38 (48.1)
Pediatric subspecialty	117 (3.9)	26 (22.4)	49 (42.2)	41 (35.3)	37 (31.9)	36 (31.0)	43 (37.1)	69 (60.0)
Pediatrics general	149 (5.0)	27 (18.1)	67 (45.0)	55 (36.9)	57 (39.0)	52 (35.6)	37 (25.3)	95 (64.2)
Physical medicine and rehabilitation	67 (2.2)	14 (20.9)	31 (46.3)	22 (32.8)	21 (31.8)	29 (43.9)	16 (24.2)	32 (47.8)
Preventative or occupational medicine	10 (0.3)	3 (30.0)	4 (40.0)	3 (30.0)	5 (50.0)	3 (30.0)	2 (20.0)	5 (50.0)
Psychiatry	259 (8.6)	46 (17.8)	98 (38.0)	114 (44.2)	84 (32.7)	102 (39.7)	71 (27.6)	113 (43.8)
Radiation oncology	33 (1.1)	4 (12.5)	8 (25.0)	20 (62.5)	5 (16.1)	15 (48.4)	11 (35.5)	20 (62.5)
Radiology	115 (3.8)	5 (4.3)	24 (20.9)	86 (74.8)	41 (35.7)	51 (44.3)	23 (20.0)	52 (46.4)
Urology	20 (0.7)	5 (25.0)	8 (40.0)	7 (35.0)	4 (20.0)	5 (25.0)	11 (55.0)	11 (55.0)
Missing	20^b^	NA	NA	NA	NA	NA	NA	NA
Time worked per week, h								
Median (IQR)	50 (40-60)	NA	NA	NA	NA	NA	NA	NA
<40	526 (17.5)	99 (19.0)	200 (38.4)	222 (42.6)	239 (45.9)	202 (38.8)	80 (15.4)	242 (46.4)
40-49	720 (24.0)	138 (19.3)	256 (35.8)	321 (44.9)	248 (34.8)	284 (39.9)	180 (25.3)	380 (53.7)
50-59	721 (24.0)	101 (14.0)	329 (45.8)	289 (40.2)	192 (26.9)	274 (38.3)	249 (34.8)	363 (50.5)
60-69	663 (22.1)	142 (21.6)	241 (36.6)	275 (41.8)	123 (18.9)	248 (38.0)	281 (43.1)	326 (49.8)
70-79	174 (5.8)	44 (25.6)	78 (45.3)	50 (29.1)	30 (17.6)	53 (31.2)	87 (51.2)	74 (43.0)
≥80	200 (6.7)	68 (34.0)	81 (40.5)	51 (25.5)	44 (22.1)	48 (24.1)	107 (53.8)	73 (36.5)
Missing	20^b^	NA	NA	NA	NA	NA	NA	NA
Primary practice setting								
Private practice	1674 (55.7)	330 (19.8)	658 (39.6)	675 (40.6)	526 (31.7)	662 (39.9)	470 (28.3)	771 (46.4)
Academic medical center	855 (28.5)	153 (18.0)	353 (41.5)	345 (40.5)	157 (18.5)	304 (35.8)	387 (45.6)	465 (54.8)
Veterans Administration hospital	78 (2.6)	17 (21.8)	35 (44.9)	26 (33.3)	35 (45.5)	18 (23.4)	24 (31.2)	46 (59.7)
Active military practice	21 (0.7)	8 (38.1)	9 (42.9)	4 (19.0)	15 (71.4)	5 (23.8)	1 (4.8)	9 (42.9)
Other	376 (12.5)	87 (23.4)	127 (34.1)	158 (42.5)	144 (39.6)	119 (32.7)	101 (27.7)	168 (46.0)
Missing	20^b^	NA	NA	NA	NA	NA	NA	NA
Compensation based on productivity, %								
None	1138 (37.8)	214 (19.0)	421 (37.4)	490 (43.6)	404 (36.0)	389 (34.7)	328 (29.3)	556 (49.6)
<25	493 (16.4)	93 (18.9)	208 (42.4)	190 (38.7)	147 (30.1)	194 (39.7)	148 (30.3)	244 (49.9)
25-50	230 (7.6)	46 (20.0)	99 (43.0)	85 (37.0)	51 (22.5)	82 (36.1)	94 (41.4)	116 (50.9)
51-75	193 (6.4)	45 (23.4)	72 (37.5)	75 (39.1)	36 (18.8)	78 (40.6)	78 (40.6)	99 (51.8)
>75	955 (31.7)	194 (20.4)	386 (40.6)	371 (39.0)	239 (25.3)	369 (39.0)	337 (35.7)	445 (46.9)
Missing	15^b^	NA	NA	NA	NA	NA	NA	NA

Abbreviations: EHR, electronic health record; NA, not applicable.

^a^ Refers to time spent on patient-related telephone calls, electronic health record inbox messages, and other work-related email.

^b^ Missing data were not included in calculations of percentages.

Vacation days taken per year varied by specialty. Among specialty disciplines with more than 30 respondents, physicians practicing anesthesiology (93 physicians [75.6%]), radiology (86 physicians [74.8%]), radiation oncology (20 physicians [62.5%]), and pathology (45 physicians [56.2%]) had the highest proportion of physicians taking more than 3 weeks of vacation, whereas family medicine (77 physicians [34.4%]), physical medicine and rehabilitation (22 physicians [32.8%]), general internal medicine (56 physicians [27.2%]), and emergency medicine (41 physicians [23.8%]) had the lowest percentage of physicians taking more than 3 weeks of vacation (Table 1).

Time spent on patient-related tasks while on vacation also varied by specialty. Among specialty disciplines with more than 30 respondents, emergency medicine (34 physicians [19.5%]), radiology (23 physicians [20.0%]), and pathology (19 physicians [24.1%]) had the lowest percentage of physicians performing 30 minutes of WOV on a typical vacation day, whereas urology (11 physicians [55.0%]), neurology (39 physicians [43.3%]), and surgery subspecialties (98 physicians [42.2%]) had the highest percentage of physicians performing 30 minutes or more of WOV on a typical vacation day (Table 1).

More than one-third of physicians indicated that each of the following was a barrier to taking vacation: finding someone to cover clinical responsibilities, the financial impact of taking vacation, and the volume of EHR inbox work to face on return to work (Table 2). On univariable analysis, the number of vacation days taken and WOV varied by gender. A lower proportion of women reported taking more than 3 weeks vacation than men (432 of 1136 women [38.0%] vs 780 of 1862 men [41.9%]). Women were also more likely than men to perform 30 minutes or more of WOV per vacation day (430 of 1131 women [38.0%] vs 555 of 1851 men [30.0%]) (eFigure in Supplement 1). On bivariable analysis, vacation days in the last year, inbox coverage, and WOV were each associated with emotional exhaustion, depersonalization, overall burnout, and professional fulfillment (Table 3 and Figure).

**Table 2.** Reported Barriers to Taking Vacation

Barrier and rating^a^	Participants, No. (%)
Finding someone to cover my clinical responsibilities	
Not at all	1391 (46.4)
A little bit	558 (18.6)
Somewhat	467 (15.6)
Quite a bit	289 (9.6)
Very much	293 (9.8)
Financial impact on my professional compensation	
Not at all	1551 (51.8)
A little bit	452 (15.1)
Somewhat	383 (12.8)
Quite a bit	303 (10.1)
Very much	308 (10.3)
The volume of electronic health record inbox work I would face on return	
Not at all	1302 (43.5)
A little bit	587 (19.6)
Somewhat	484 (16.2)
Quite a bit	318 (10.6)
Very much	299 (10.0)

^a^ Participants were asked, “Rate how much of a barrier each of the following is to taking vacation for you.”

**Table 3.** Bivariable Analysis of Vacation Characteristics and Measures of Burnout and Professional Fulfillment

Questions and responses	Emotional exhaustion^a^		Depersonalization^a^		Overall burnout^b^		Professional fulfillment^c^	
	Score, mean (SD)	*P* value	Score, mean (SD)	*P* value	Score, mean (SD)	*P* value	Score, mean (SD)	*P* value
How many days of vacation (workdays) did you take in the last 12 mo?								
None	22.63 (15.09)	<.001	6.98 (7.03)	<.001	93 (45.4)	<.001	6.21 (2.59)	<.001
1-5	23.00 (13.09)		6.41 (6.40)		166 (43.3)		6.34 (2.14)	
6-10	22.14 (12.95)		6.77 (6.39)		226 (42.6)		6.28 (2.22)	
11-15	21.37 (12.36)		6.29 (6.10)		260 (39.8)		6.59 (1.98)	
16-20	20.25 (12.76)		5.77 (5.93)		189 (35.0)		6.67 (2.13)	
>20	18.54 (12.53)		5.35 (5.80)		210 (31.5)		6.76 (2.26)	
Do you have full EHR inbox coverage while on vacation?								
Yes	19.72 (12.66)	<.001	5.82 (5.96)	.007	504 (34.5)	<.001	6.64 (2.19)	.003
No	22.22 (13.08)		6.44 (6.35)		640 (42.4)		6.41 (2.17)	
On a typical vacation day in the last year, how much time (minutes) did you spend responding to patient-related phone calls, inbox messages in the EHR, and on other work-related email?								
None	18.67 (13.06)	<.001	5.72 (5.99)	<.001	289 (33.1)	<.001	6.60 (2.25)	<.001
1-30	19.91 (12.12)		5.82 (5.92)		380 (34.3)		6.68 (2.02)	
30-60	23.30 (12.58)		6.40 (6.15)		242 (44.6)		6.27 (2.11)	
60-90	25.47 (12.97)		7.47 (6.75)		116 (53.2)		6.23 (2.24)	
>90	25.45 (14.25)		7.51 (7.46)		113 (51.4)		6.26 (2.62)	

Abbreviation: EHR, electronic health record.

^a^ Assessed using the full-length emotional exhaustion and depersonalization domains of the Maslach Burnout Inventory. Per the standard scoring of the Maslach Burnout Inventory for health care workers, physicians with scores on the emotional exhaustion subscale of 27 or more or on the depersonalization subscale of 10 or more are considered to have a score in that dimension.

^b^ Respondents were considered to have symptoms of burnout if they had a high emotional exhaustion and/or high depersonalization scores using the Maslach Burnout Inventory.

^c^ The Stanford Professional Fulfillment Index was used to measure professional fulfillment, with scores normalized on a scale of 0 to 10 (higher scores are more favorable).

**Figure.**
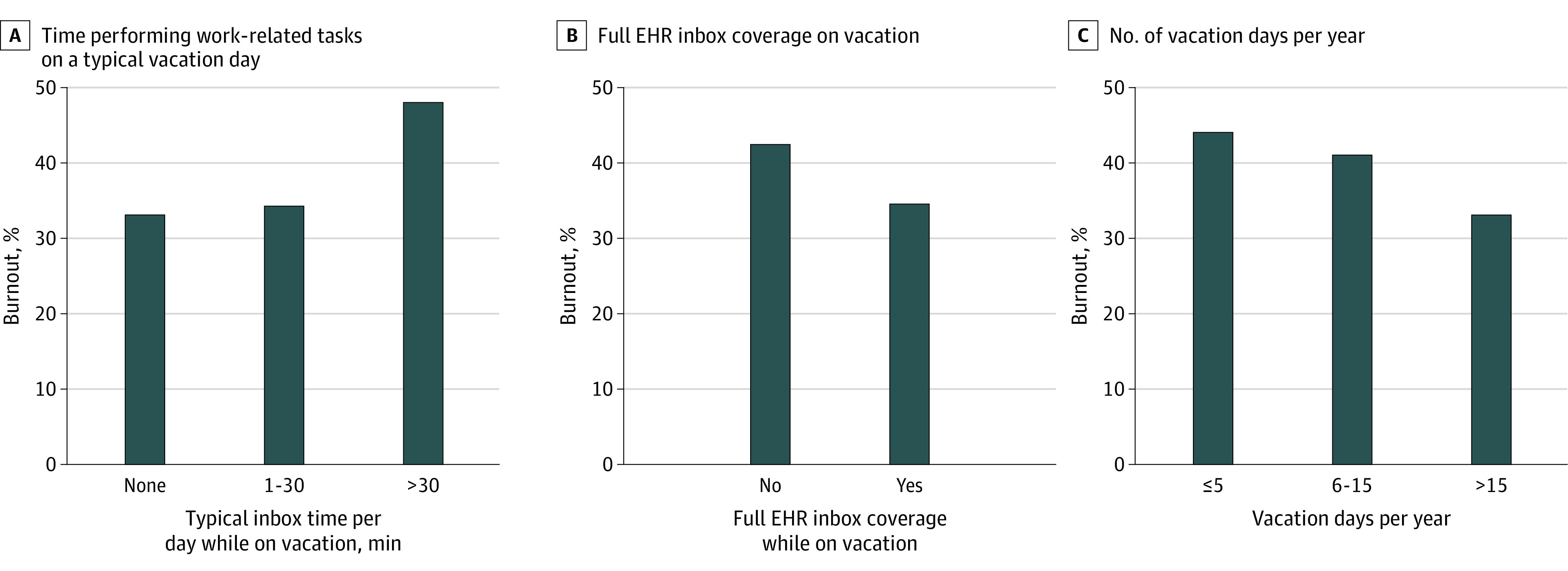
Personal and Institutional Vacation Behaviors and Prevalence of Burnout Graphs show burnout rates in relation to time performing work-related tasks on a typical vacation day (A), full electronic health record (EHR) inbox coverage during vacation (B), and number of vacation days per year (C).

We next conducted multivariable analysis to identify factors associated with taking more than 3 weeks of vacation per year. After controlling for personal and professional characteristics, relationship status (odds ratio [OR], 1.41 [95% CI, 1.04-1.92] for being married) was associated with taking more than 3 weeks of vacation. Concern about finding someone to cover clinical responsibilities (OR, 0.48 [95% CI, 0.35-0.65] for quite a bit; OR, 0.30 [95% CI, 0.21-0.43] for very much) and financial concerns (OR, 0.49 [95% CI, 0.36-0.66] for quite a bit; OR, 0.38 [95% CI, 0.27-0.54] for very much) were associated with decreased likelihood of taking more than 3 weeks of vacation per year. Hours worked per week (OR, 0.99 [95% CI, 0.99-1.00] for each additional hour) was associated with lower odds of taking more than 3 weeks of vacation. Working in a Veterans Administration hospital (OR, 0.57 [95% CI, 0.33-0.97]) or active military setting (OR, 0.26 [95% CI, 0.07-0.76]) was associated with lower odds of taking more than 3 weeks of vacation compared with private practice. Age, gender, and having children were not associated with taking more than 3 weeks of vacation after adjusting for other personal and professional factors (Table 4).

**Table 4.** Multivariable Analysis of Factors Associated With the Outcome of Taking More than 15 Vacation Days per Year^a^

Factor	OR (95% CI)	*P* value
Age, y		
<35	1 [Reference]	NA
35-44	0.89 (0.57-1.41)	.62
45-54	0.86 (0.53-1.40)	.53
55-64	0.82 (0.50-1.38)	.46
≥65	0.96 (0.56-1.67)	.89
Gender		
Male	1 [Reference]	NA
Female	0.90 (0.74-1.09)	.28
Relationship status		
Single	1 [Reference]	NA
Married	1.41 (1.04-1.92)	.03
Partnered	1.41 (0.89-2.24)	.14
Widowed	1.24 (0.46-3.22)	.67
Age of youngest child, y		
No children	1 [Reference]	NA
<5	0.93 (0.66-1.31)	.67
5-12	1.30 (0.94-1.79)	.11
13-18	1.01 (0.71-1.43)	.96
19-22	1.22 (0.83-1.78)	.31
>23	1.20 (0.85-1.71)	.30
Compensation based on productivity, %		
None	1 [Reference]	NA
<25	0.98 (0.77-1.26)	.89
25-50	0.99 (0.71-1.38)	.97
51-75	0.98 (0.68-1.41)	.91
>75	1.03 (0.82-1.28)	.82
Work hours (each additional hour per week)	0.99 (0.99-1.00)	.005
Practice setting		
Private practice	1 [Reference]	NA
Academic medical center	0.91 (0.74-1.13)	.40
Veterans Administration hospital	0.57 (0.33-0.97)	.04
Active military practice	0.26 (0.07-0.76)	.02
Other	1.02 (0.78-1.33)	.90
Inbox coverage		
No	1 [Reference]	NA
Yes	1.07 (0.90-1.26)	.45
Concern regarding finding someone to cover clinical responsibilities		
None or a little bit	1 [Reference]	NA
Moderate	0.77 (0.61-0.99)	.04
Quite a bit	0.48 (0.35-0.65)	<.001
Very much	0.30 (0.21-0.43)	<.001
Financial concerns		
None or a little bit	1 [Reference]	NA
Moderate	0.71 (0.55-0.93)	.01
Quite a bit	0.49 (0.36-0.66)	<.001
Very much	0.38 (0.27-0.54)	<.001
Concern about inbox volume on return		
None or a little bit	1 [Reference]	NA
Moderate	1.11 (0.88-1.41)	.38
Quite a bit	0.77 (0.57-1.03)	.08
Very much	0.96 (0.68-1.34)	.81
Specialty		
Internal medicine subspecialty	1 [Reference]	
Anesthesiology	5.41 (3.24-9.25)	<.001
Dermatology	1.93 (1.05-3.57)	.03
Emergency medicine	0.45 (0.28-0.72)	<.001
Family medicine	0.93 (0.63-1.39)	.74
General internal medicine	0.74 (0.48-1.12)	.15
General pediatrics	0.84 (0.54-1.32)	.45
General surgery	1.40 (0.84-2.32)	.20
General surgery subspecialty	1.09 (0.74-1.61)	.65
Neurology	1.30 (0.76-2.20)	.33
Neurosurgery	1.00 (0.43-2.21)	.99
Obstetrics and gynecology	1.83 (1.14-2.94)	.01
Ophthalmology	1.08 (0.67-1.74)	.75
Orthopedic surgery	1.41 (0.89-2.24)	.14
Other	0.95 (0.62-1.45)	.81
Otolaryngology	0.72 (0.22-2.03)	.56
Pathology	2.16 (1.25-3.79)	.01
Pediatric subspecialty	0.78 (0.48-1.27)	.32
Physical medicine and rehabilitation	1.03 (0.55-1.89)	.93
Preventive medicine or occupational medicine	0.68 (0.14-2.73)	.60
Psychiatry	1.28 (0.87-1.89)	.20
Radiation oncology	2.71 (1.17-6.60)	.02
Radiology	4.62 (2.74-8.01)	<.001
Urology	0.88 (0.31-2.32)	.81

Abbreviations: NA, not applicable; OR, odds ratio.

^a^ Model included the following variables: age category (<35 years old referent), gender (male referent), relationship status (single referent), age of youngest child (no children referent), compensation based on productivity (vs none), hours worked per week (each additional hour), practice setting (private practice referent category), inbox coverage (vs no), concern regarding finding someone to cover clinical responsibilities (vs none or a little bit), financial concerns (vs none or a little bit), concern about inbox volume on return (vs none or a little bit), and specialty (internal medicine subspecialty referent category).

Next, we conducted an exploratory multivariable analysis using the same variables to identify factors associated with WOV. Having full EHR inbox coverage while on vacation, which was available to slightly less than one-half of physicians (Table 1), was independently associated with lower odds of WOV (OR, 0.68 [95% CI, 0.57-0.80]). Female gender (OR, 1.62 [95% CI, 1.33-1.97]), working in an academic medical center (OR, 2.25 [95% CI, 1.83-2.78]), age 55 to 64 years (OR, 1.77 [95% CI, 1.03-3.10]), and age of youngest child 5 to 12 years (OR, 1.51 [95% CI, 1.09-2.10]) were associated with higher odds of WOV. In addition, each additional hour worked per week (OR, 1.03 [95% CI, 1.03-1.04]) was associated with higher odds of WOV (eTable 1 in Supplement 1).

We next conducted a multivariable analysis using the same variables to identify factors associated with burnout. In this analysis, taking more than 3 weeks of vacation per year (OR, 0.66 [95% CI, 0.45-0.98] for 16-20 days; OR, 0.59 [95% CI, 0.40-0.86] for >20 days vs none) and having full EHR inbox coverage while on vacation (OR, 0.74 [95% CI, 0.63-0.88]) were associated with lower risk of burnout, whereas spending 30 minutes or more on WOV on a typical vacation day (OR, 1.58 [95% CI, 1.22-2.04] for 30-60 minutes; OR, 1.97 [95% CI, 1.41-2.77] for 60-90 minutes; OR, 1.92 [95% CI, 1.36-2.73] for >90 minutes) was associated with higher risk of burnout (eTable 2 in Supplement 1). Finally, on multivariable analysis including the same variables to identify factors associated with professional fulfillment, full EHR inbox coverage while on vacation was associated with higher likelihood of professional fulfillment (OR, 1.32 [95% CI, 1.13-1.56]) (eTable 3 in Supplement 1).

## Discussion

To our knowledge, this cross-sectional study is the first large study of vacation behaviors among US physicians and the first to evaluate associations of vacation behaviors with burnout. Notably, more than one-half of US physicians (59.6%) take 3 or fewer weeks of vacation per year, with 1 in 5 taking 5 or fewer days. Concerns about finding cross-coverage for clinical responsibilities and concerns about the financial impact of taking vacation (eg, by not meeting productivity targets or continued overhead costs) are associated with taking less than 3 weeks of vacation. The majority of physicians (70.4%) performed WOV on a typical vacation day, with one-third working 30 minutes or more per vacation day. On multivariable analysis controlling for personal and professional factors, women were more likely to perform more than 30 minutes of WOV, consistent with previous reports that women have more after-hours work.^21^ In multivariable analysis, working in the Veterans Administration or an active military setting was associated with lower odds of taking more than 3 weeks vacation, whereas working in an academic setting was associated with more than twice the odds of WOV.

Taking less vacation and performing WOV was associated with higher mean emotional exhaustion and depersonalization scores and lower professional fulfillment scores. After adjusting for other key factors (age, gender, relationship status, children, specialty, and practice setting), the number of vacation days taken and having full EHR inbox coverage while on vacation were associated with lower odds of burnout, whereas spending 30 minutes or more on WOV on a typical vacation day was associated with higher odds of burnout. Having full EHR inbox coverage while on vacation was also associated with higher odds of professional fulfillment.

Extensive evidence indicates many physicians are experiencing chronic work overload. Physicians work more hours per work week than the general US working population, with the typical full-time physician working 54 hours per week, averaging 10 hours more than other US workers each week.^32^ Less than 10% of US workers in other fields (9.2%) reported working 55 hours or more per week, compared with 40.7% of physicians.^32^ Longer work hours per week are associated with higher rates of burnout among physicians, with approximately 2% increase in odds of burnout for each extra hour worked per week.^26^ In addition, working long hours may impact workers’ physical health: working 55 hours or more week is associated with higher rates of heart disease and stroke.^33^

Our current data suggest that most physicians are taking 3 or fewer weeks of vacation per year and not fully disconnecting while on vacation. These vacation behaviors are factors likely to exacerbate the effects of chronic work overload associated with long work hours and further heighten the risk of burnout. This chronic work overload is associated with higher rates of burnout and lower professional fulfillment. Occupational burnout among physicians is a serious threat to patient and physician well-being and health system goals and has been associated with higher rates of medical errors,^34^ longer hospital stays, greater mortality, lower rates of patient satisfaction,^35,36^ reduction in work hours,^37^ turnover,^35,38^ excess health care costs,^39,40,41^ and physical and mental health issues for physicians.^42,43,44^

The fact that two-thirds of physicians are obligated to continue to provide clinical care to their patients while on vacation should be considered a marker of poorly designed systems of teamwork, inadequate clinical staffing, and poorly designed cross-coverage systems. Previous research^17^ suggests that physicians’ ability to disconnect from work is associated with lower burnout but is also associated with lower patient satisfaction. Without system strategies, this situation puts physicians in a double bind. Patient care needs predictably continue even while a physician is on vacation, and it is incumbent on organizations to build systems to provide EHR inbox coverage and deliver team-based care to meet patient needs when a physician is out of office. In this regard, the inability of most physicians to completely disengage from patient care while on vacation should be considered a system failure—one with consequences for both the patient and the physician.

Suboptimal vacation behaviors, such as fewer days of vacation taken and more time spent providing patient care while on vacation, may also be harmful to physicians’ personal relationships. Relationships require time together to be nurtured and strengthened; vacation has been shown to enhance personal relationships in the general population.^8^ Among physician spouses, time spent awake together with their physician partner is the primary factor associated with relationship satisfaction.^45^ A lack of attention to one’s personal relationships may be harmful to both patient care (more patient complaints) and physicians (more occupational distress and burnout).^46^

Given the high prevalence of burnout among physicians, efforts to mitigate this reduced time for recovery and restoration are warranted. According to surveys by the American Medical Association^47^ and the Physicians Advocacy Institute,^48^ the majority of physicians are employed, a trend that is expected to continue. For example, Merritt Hawkins reported in 2022 that more than 90% of physicians accepting new positions will practice as employees and not as independent practice owners or partners.^49^ Our results suggest that ensuring physicians take at least 3 weeks of vacation per year and providing coverage for clinical work, including full EHR inbox coverage while physicians are on vacation, may be tangible and pragmatic organizational actions to mitigate burnout risk. Overt efforts to track, normalize, and model taking vacation would likely be helpful. For example, organizations could track the percentage of allotted vacation days taken and amount of patient care work done while on vacation in organizational dashboards as leading indicators of systemic stress and occupational burnout. In addition, our data suggest that routinely providing full clinical and EHR inbox coverage for physicians while on vacation and reducing the financial incentives that encourage physicians to forgo vacation (eg, overreliance on relative value unit–related incentive-based compensation) may increase uptake of vacation time and fully disconnecting from work while on vacation.

### Limitations

Our study is subject to limitations. Although we studied a large sample of US physicians, and the results of a secondary survey suggested participants were representative of US physicians, the participation rate was low and response bias remains a concern. It is unknown whether physicians who take fewer days of vacation or who spend more time on WOV are more or less likely to participate in a survey assessing these dimensions. This is a cross-sectional study, so causation cannot be determined. We asked about the number of vacation days per year and do not know whether some respondents, particularly those who are self-employed, included within this estimate paid time off for other purposes, such as illness, family medical leave, or attending professional meetings. Academic physicians vary widely in day-to-day activities and may have personal preferences for flexibility in scheduling multiple work responsibilities that cannot be accounted for in our analyses. The overall sample size for some specialties was modest, which limits precision when comparing vacation behaviors across specialties.

## Conclusions

In this cross-sectional study of 3024 physicians, vacation behaviors were associated with physician well-being. Specifically, the number of vacation days taken per year and time spent performing patient-care work tasks while on vacation were associated with physician burnout. Where not currently in place, institutional efforts to provide operational support and clinical coverage, including full EHR inbox coverage, when physicians are on vacation should be pursued. Normalizing the expectation that physicians take time off and fully disconnect from clinical work while away may also be beneficial at both the organizational and professional level. Such efforts may be critical and tangible system-based approaches to mitigate the high rates of occupational burnout among physicians.

## Notes

Supplement 1. **eAppendix.** Supplemental Methods **eTable 1.** Multivariable Analysis of Factors Associated with Performing Patient-Related Care on Vacation^a^ **eTable 2.** Multivariable Analysis of Factors Associated With Burnout^a^ **eTable 3.** Multivariable Analysis of Factors Associated With Professional Fulfillment^a^ **eFigure.** Patient-Related Tasks (≥ 30 min) on a Typical Vacation Day by Gender
eReferences

Supplement 2.
Data Sharing Statement
