# Supplementary material for: Vacation Days Taken, Work During Vacation, and Burnout Among US Physicians
Source: JAMA Netw Open. 2024 Jan 12;7(1):e2351635. doi: 10.1001/jamanetworkopen.2023.51635 (PMC10787314; doi:10.1001/jamanetworkopen.2023.51635)
Supplement: Supplement 1. — eAppendix. Supplemental Methods eTable 1. Multivariable Analysis of Factors Associated with Performing Patient-Related Care on Vacationa eTable 2. Multivariable Analysis of Factors Associated With Burnouta eTable 3. Multivariable Analysis of Factors Associated With Professional Fulfillmenta eFigure. Patient-Related Tasks (≥ 30 min) on a Typical Vacation Day by Gender eReferences [file jamanetwopen-e2351635-s001.pdf]

Supplemental Online Content

Sinsky CA, Trockel MT, Dyrbye LN, et al. Vacation days taken, work during vacation, and burnout among US physicians. *JAMA Netw Open*. 2024;7(1):e2351635. doi:10.1001/jamanetworkopen.2023.51635

eAppendix. Supplemental Methods
eTable 1. Multivariable Analysis of Factors Associated with Performing Patient-Related Care on Vacation[a]
eTable 2. Multivariable Analysis of Factors Associated With Burnout[a]
eTable 3. Multivariable Analysis of Factors Associated With Professional Fulfillment[a]
eFigure. Patient-Related Tasks (≥ 30 min) on a Typical Vacation Day by Gender
eReferences

This supplemental material has been provided by the authors to give readers additional information about their work.

© 2024 Sinsky CA et al. *JAMA Network Open.*

eAppendix. Supplemental Methods

Study Participants

The survey methodology used in this study has been previously reported.[1] A sample of 4,000 US physicians drawn from the American Medical Association (AMA) Physician Professional Database (PPD), previously known as the AMA Physician Masterfile, with oversampling of physicians in fields outside of internal medicine, family medicine, general pediatrics and OB/GYN, was mailed paper surveys along with a $20 incentive check on November 16, 2020. Of these 329 were returned as undeliverable, resulting in a final sample of 3,671 physicians. Non-responders were sent a second mailing on December 8, 2020. In total, 1162 paper surveys (31.7%) were returned by March 26,2021 and included in the analysis. Concurrently a second representative sample of 90,000 physicians, also drawn from the AMA Physician Masterfile, was emailed an on-line version of the survey on November 16, 2020, with follow up invitations to non-responders over the next several weeks. Responders to both the paper and electronic surveys were similar in terms of age, gender, years in practice and symptoms of burnout. Both sets of responses were pooled for analysis. Half of survey recipients were randomly assigned to receive a sub-survey consisting of six vacation items.

Study measures

Burnout in physicians was assessed using the full-length emotional exhaustion and depersonalization scales of the Maslach Burnout Inventory (MBI)[2-4].

Professional fulfillment was measured using the professional fulfillment scale from the Stanford Professional Fulfillment Index.[5] The professional fulfillment sub-scale includes 6 items that assess professional satisfaction, self-worth at work, self-efficacy, happiness, and meaning in work. Participants indicate how accurately each item reflects their experience over the last two weeks on a five-point scale

© 2024 Sinsky CA et al. *JAMA Network Open.*

with response options ranging from "not at all true" (0 points) to "completely true" (4 points). Scores from the individual items are aggregated and normed to a scale of 0 to 10 (higher more favorable).

Six vacation items were constructed for this study, investigating the number of days of vacation taken in the last 12 months, whether full EHR coverage is available while on vacation, how much time is spent performing patient-related tasks on a typical vacation day, and inquiring about barriers to taking vacation.

How vacation time is defined and allocated for physicians varies greatly across practice setting. Using the definition of vacation that applies to your practice, please answer the following questions.

How many days of vacation did you take in the last 12 months?

- ◯ None (1)
- ◯ 1-5 work days (about 1 week) (2)
- ◯ 6-10 work days (3)
- ◯ 11-15 work days (4)
- ◯ 16-20 work days (5)
- ◯ >20 work days (6)

Do you have full EHR inbox coverage (i.e., for patient-related electronic messages such as those via a patient portal) while on vacation?

- ◯ Yes (1)
- ◯ No (0)

© 2024 Sinsky CA et al. *JAMA Network Open.*

On a typical vacation day in the last year, how much time did you spend responding to patient-related phone calls, inbox messages in the EHR, and on other work-related email

- None (1)
- 1-30 minutes (2)
- 30-60 minutes (3)
- 60-90 minutes (4)
- >90 minutes (5)

© 2024 Sinsky CA et al. *JAMA Network Open.*

Rate how much of a barrier each of the following is to taking vacation for you.

	Not at all (1)	A little bit (2)	Somewhat (3)	Quite a bit (4)	Very much (5)
Finding someone to cover my clinical responsibilities (e.g., urgent patient care needs, patient calls, EHR inbox) (1)	○	○	○	○	○
Financial impact on my professional compensation (i.e., reduced wRVUs, not meeting productivity targets, continued overhead costs) (2)	○	○	○	○	○
The volume of EHR inbox work I would face on my return (3)	○	○	○	○	○

© 2024 Sinsky CA et al. *JAMA Network Open.*

eTable 1: Multivariable Analysis of Factors Associated with Performing Patient-Related Care on Vacation[a]

Outcome	Predictor	OR (95% CI)	P-value
On a typical vacation day in the last year, how much time (min) did you spend responding to patient-related phone calls, inbox messages in the EHR, and on other work-related email? (>30min)	Age (vs. age <35)		
	35-44	1.38 (0.85-2.3)	0.20
	45-54	1.49 (0.89-2.55)	0.14
	**55-64**	**1.77 (1.03-3.1)**	**0.04**
	≥65	1.32 (0.73-2.41)	0.37
	**Gender (vs male)**		
	**Female**	**1.62 (1.33-1.97)**	**<0.001**
	Relationship Status (vs single)		
	Married	0.93 (0.69-1.27)	0.65
	Partnered	1.04 (0.64-1.67)	0.87
	Widowed/widower	1.68 (0.62-4.4)	0.30
	Age of youngest child (vs no children)		
	<5	1.09 (0.76-1.55)	0.64
	**5-12**	**1.51 (1.09-2.1)**	**0.01**
	13-18	1.33 (0.93-1.89)	0.12
	19-22	1.3 (0.88-1.92)	0.19
	>23	1 (0.69-1.45)	>0.99
	Compensation based on productivity (vs none)		
	<25%	0.77 (0.59-1)	0.05
	**25-50%**	**1.39 (1-1.94)**	**0.049**
	51-75%	1.24 (0.86-1.77)	0.25
	**>75%**	**1.42 (1.13-1.78)**	**0.002**
	**Work Hours (each additional hour per week)**	**1.03 (1.03-1.04)**	**<0.001**
	**Practice setting (vs private practice)**		
	**Academic medical center**	**2.25 (1.83-2.78)**	**<0.001**
	Veterans hospital	1.39 (0.79-2.38)	0.24
	Active military practice	0.14 (0.01-0.68)	0.06
	Other	1.17 (0.88-1.56)	0.28
	Specialty (vs. IM subspecialty)		
	**Anesthesiology**	**0.5 (0.3-0.82)**	**0.01**
	Dermatology	0.87 (0.46-1.61)	0.65
	**Emergency medicine**	**0.57 (0.35-0.93)**	**0.03**
	Family medicine	1.28 (0.87-1.89)	0.21
	General IM	0.82 (0.55-1.23)	0.34
	General Pediatrics	0.78 (0.48-1.26)	0.31
	**General surgery**	**0.44 (0.25-0.75)**	**0.003**
	General surgery subspecialty	0.74 (0.5-1.09)	0.13
	Neurology	1.08 (0.63-1.83)	0.78
	Neurosurgery	0.63 (0.27-1.42)	0.27
	Obstetrics and gynecology	0.63 (0.38-1.01)	0.06
	**Ophthalmology**	**0.53 (0.31-0.87)**	**0.01**

© 2024 Sinsky CA et al. *JAMA Network Open.*

	Orthopedic surgery	0.69 (0.43-1.11)	0.13
	Other	0.85 (0.55-1.3)	0.45
	Otolaryngology	0.55 (0.18-1.55)	0.27
	**Pathology**	**0.4 (0.21-0.74)**	**0.004**
	**Pediatric subspecialty**	**0.61 (0.37-0.99)**	**0.048**
	PM & R	0.52 (0.26-0.98)	0.05
	Preventive medicine/occupational medicine	0.26 (0.01-1.53)	0.21
	Psychiatry	0.71 (0.47-1.06)	0.10
	Radiation oncology	0.87 (0.35-2.04)	0.75
	**Radiology**	**0.46 (0.26-0.79)**	**0.01**
	Urology	1.93 (0.73-5.3)	0.19
	**Having full EHR coverage while on vacation (vs. no)**	**0.68 (0.57-0.8)**	**<0.001**

[a]Model included the following variables: Age (< 35 years old referent), gender (male referent), relationship status (single referent), age of youngest child (no children referent), Compensation based on productivity (vs none), hours worked per week (each additional hour), practice setting (private practice referent category), specialty (internal medicine subspecialty referent category), and having full EHR coverage while on vacation.

Note: Boldface indicates statistical significance (p<0.05).

© 2024 Sinsky CA et al. *JAMA Network Open.*

eTable 2: Multivariable Analysis of Factors Associated With Burnout[a]

Outcome	Predictor	OR (95% CI)	P-value
**Burnout**	Age (vs. age <35)		
	35-44	0.91 (0.59-1.41)	0.68
	45-54	0.86 (0.54-1.37)	0.53
	55-64	0.76 (0.47-1.24)	0.28
	**≥65**	**0.38 (0.22-0.64)**	**<0.001**
	Gender (vs male)		
	Female	1.17 (0.97-1.41)	0.10
	Relationship Status (vs single)		
	Married	0.77 (0.58-1.02)	0.07
	Partnered	0.75 (0.48-1.18)	0.22
	Widowed/widower	1.10 (0.40-2.92)	0.85
	Age of youngest child (vs no children)		
	<5	0.83 (0.60-1.15)	0.25
	5-12	0.93 (0.69-1.27)	0.66
	**13-18**	**0.71 (0.51-0.99)**	**0.04**
	19-22	0.76 (0.52-1.11)	0.16
	>23	0.81 (0.57-1.15)	0.24
	Compensation based on productivity (vs none)		
	<25%	1.15 (0.90-1.48)	0.25
	25-50%	1.00 (0.72-1.38)	0.99
	51-75%	1.00 (0.70-1.43)	0.99
	>75%	1.10 (0.89-1.37)	0.37
	**Work Hours per week (each additional hour)**	**1.02 (1.01-1.03)**	**<0.001**
	Practice setting (vs private practice)		
	**Academic medical center**	**0.76 (0.62-0.94)**	**0.01**
	Veterans hospital	0.60 (0.34-1.02)	0.06
	Active military practice	0.62 (0.22-1.57)	0.33
	Other	0.99 (0.75-1.30)	0.95
	**Inbox coverage (vs no)**		
	**Yes**	**0.74 (0.63-0.88)**	**<0.001**
	**How many days of vacation did you take in the last 12 months? (workdays) (vs.None)**		
	1-5	0.74 (0.5-1.11)	0.14
	6-10	0.78 (0.53-1.14)	0.19
	11-15	0.8 (0.55-1.16)	0.23
	**16-20**	**0.66 (0.45-0.98)**	**0.04**
	**>20**	**0.59 (0.4-0.86)**	**0.01**
	**On a typical vacation day in the last year, how much time (min) did you spend responding to patient-related phone calls, inbox messages in the EHR, and on other work-related email? (vs. none)**		

© 2024 Sinsky CA et al. *JAMA Network Open.*

	1-30	1.04 (0.84-1.29)	0.71
	**30-60**	**1.58 (1.22-2.04)**	**<0.001**
	**60-90**	**1.97 (1.41-2.77)**	**<0.001**
	**>90**	**1.92 (1.36-2.73)**	**<0.001**
	Specialty (vs. IM subspecialty)		
	Anesthesiology	1.28 (0.79-2.07)	0.32
	Dermatology	0.98 (0.53-1.80)	0.95
	**Emergency medicine**	**2.41 (1.56-3.74)**	**<0.001**
	**Family medicine**	**1.56 (1.06-2.29)**	**0.02**
	General IM	1.18 (0.79-1.75)	0.42
	General Pediatrics	0.93 (0.59-1.47)	0.77
	General surgery	0.71 (0.42-1.18)	0.19
	**General surgery subspecialty**	**0.52 (0.35-0.77)**	**0.001**
	Neurology	1.29 (0.76-2.19)	0.34
	Neurosurgery	0.47 (0.19-1.09)	0.09
	Obstetrics and gynecology	1.14 (0.70-1.83)	0.60
	Ophthalmology	0.83 (0.51-1.36)	0.47
	Orthopedic surgery	0.77 (0.48-1.24)	0.29
	Other	0.82 (0.53-1.25)	0.35
	Otolaryngology	0.78 (0.26-2.11)	0.63
	**Pathology**	**0.32 (0.15-0.64)**	**0.002**
	**Pediatric subspecialty**	**0.59 (0.35-0.98)**	**0.045**
	PM & R	0.88 (0.47-1.60)	0.67
	Preventive medicine/occupational medicine	0.65 (0.09-2.96)	0.61
	Psychiatry	1.40 (0.95-2.05)	0.09
	Radiation oncology	0.87 (0.35-2.06)	0.76
	Radiology	1.15 (0.69-1.88)	0.59
	Urology	1.06 (0.40-2.76)	0.90

[a]Model included the following variables: Age (< 35 years old referent), gender (male referent), relationship status (single referent) age of youngest child (no children referent), Compensation based on productivity (vs none), hours worked per week (each additional hour), practice setting (private practice referent category), inbox coverage (vs no), days of workday vacation days (vs none), time on patient-related tasks on vacation (vs none), specialty (internal medicine subspecialty referent category).

Note: Boldface indicates statistical significance (p<0.05).

© 2024 Sinsky CA et al. *JAMA Network Open.*

eTable 3: Multivariable Analysis of Factors Associated With Professional Fulfillment[a]

Outcome	Predictor	OR (95% CI)	P-value
**Professional Fulfillment**	Age (vs. age <35)		
	35-44	1.12 (0.71-1.77)	0.64
	45-54	1.15 (0.71-1.88)	0.57
	55-64	0.99 (0.6-1.66)	0.98
	**≥65**	**1.9 (1.11-3.28)**	**0.02**
	Gender (vs male)		
	**Female**	**0.78 (0.65-0.94)**	**0.01**
	Relationship Status (vs single)		
	Married	1.1 (0.82-1.47)	0.52
	Partnered	1.03 (0.65-1.63)	0.89
	Widowed/widower	1.08 (0.4-2.87)	0.87
	Age of youngest child (vs no children)		
	<5	1.24 (0.89-1.72)	0.21
	5-12	1.06 (0.78-1.45)	0.70
	13-18	1.28 (0.91-1.79)	0.15
	19-22	1.38 (0.96-2.01)	0.08
	**>23**	**1.48 (1.05-2.08)**	**0.02**
	Compensation based on productivity (vs none)		
	<25%	0.86 (0.67-1.1)	0.23
	25-50%	1.12 (0.81-1.53)	0.48
	51-75%	0.99 (0.7-1.39)	0.95
	>75%	1.05 (0.85-1.3)	0.64
	**Work Hours per week (each additional hour)**	**0.99 (0.99-1)**	**0.01**
	Practice setting (vs private practice)		
	Academic medical center	1.02 (0.83-1.24)	0.89
	Veterans hospital	0.63 (0.36-1.07)	0.10
	Active military practice	0.92 (0.34-2.32)	0.87
	Other	0.97 (0.74-1.26)	0.81
	**Inbox coverage (vs no)**		
	**Yes**	**1.32 (1.13-1.56)**	**<0.001**
	How many days of vacation did you take in the last 12 months? (workdays) (vs.None)		
	1-5	1.02 (0.7-1.49)	0.57
	6-10	1.15 (0.86-1.53)	0.91
	11-15	1.06 (0.82-1.37)	0.85
	16-20	1.07 (0.82-1.4)	0.81
	>20	1.22 (0.94-1.58)	0.36
	On a typical vacation day in the last year, how much time (min) did you spend responding to patient-related phone calls, inbox messages in the EHR, and on other work-related email? (vs. none)		

© 2024 Sinsky CA et al. *JAMA Network Open.*

	1-30	0.9 (0.74-1.1)	0.31
	30-60	0.78 (0.61-1.01)	0.06
	60-90	0.85 (0.6-1.19)	0.34
	>90	1.13 (0.8-1.58)	0.49
	Specialty (vs. IM subspecialty)		
	Anesthesiology	1.01 (0.62-1.63)	0.96
	**Dermatology**	**1.81 (1-3.26)**	**0.048**
	Emergency medicine	0.78 (0.49-1.22)	0.28
	Family medicine	1.09 (0.73-1.6)	0.68
	General IM	1.26 (0.85-1.87)	0.26
	General Pediatrics	1.02 (0.65-1.6)	0.92
	General surgery	1.62 (0.99-2.65)	0.05
	**General surgery subspecialty**	**1.85 (1.27-2.69)**	**0.001**
	**Neurology**	**1.78 (1.06-2.97)**	**0.03**
	**Neurosurgery**	**2.93 (1.34-6.54)**	**0.01**
	**Obstetrics and gynecology**	**1.77 (1.11-2.82)**	**0.02**
	**Ophthalmology**	**2.43 (1.54-3.88)**	**<0.001**
	Orthopedic surgery	1.22 (0.77-1.92)	0.40
	Other	1.07 (0.7-1.61)	0.76
	**Otolaryngology**	**3.19 (1.21-8.98)**	**0.02**
	Pathology	1.62 (0.94-2.79)	0.08
	Pediatric subspecialty	1.28 (0.78-2.08)	0.32
	PM & R	0.81 (0.42-1.51)	0.52
	Preventive medicine/occupational medicine	0.82 (0.16-3.32)	0.79
	Psychiatry	1.4 (0.96-2.04)	0.08
	**Radiation oncology**	**2.52 (1.1-5.95)**	**0.03**
	Radiology	0.85 (0.51-1.4)	0.54
	Urology	1.02 (0.36-2.66)	0.97

[a]Model included the following variables: Age (< 35 years old referent), gender (male referent), relationship status (single referent) age of youngest child (no children referent), Compensation based on productivity (vs none), hours worked per week (each additional hour), practice setting (private practice referent category), inbox coverage (vs no), days of workday vacation days (vs none), time on patient-related tasks on vacation (vs none), specialty (internal medicine subspecialty referent category).

Note: Boldface indicates statistical significance (p<0.05).

© 2024 Sinsky CA et al. *JAMA Network Open.*

eFigure: Patient-Related Tasks (≥ 30 min) on a Typical Vacation Day by Gender

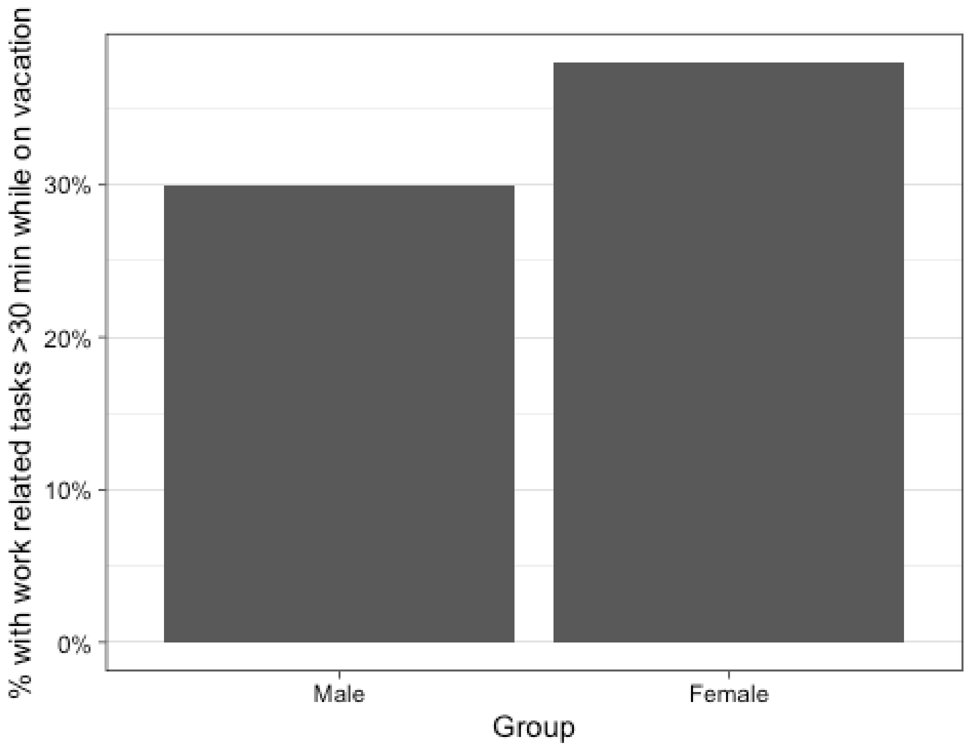

© 2024 Sinsky CA et al. *JAMA Network Open.*

eReferences

1. Shanafelt TD, West CP, Sinsky C, et al. Changes in Burnout and Satisfaction With Work-Life Integration in Physicians and the General US Working Population Between 2011 and 2020. *Mayo Clinic proceedings.* 2022;97(3):491-506.
2. Maslach C, Jackson SE, Leiter MP. *Maslach burnout inventory manual.* Palo Alto, Calif. (577 College Ave., Palo Alto 94306); Palo Alto, Calif: Consulting Psychologists Press; 1996.
3. Leiter MP, Durup J. The discriminant validity of burnout and depression: A confirmatory factor analytic study. *Anxiety, Stress, & Coping.* 1994;7(4):357-373.
4. Rafferty JP, Lemkau JP, Purdy RR, Rudisill JR. Validity of the Maslach Burnout Inventory for family practice physicians. *Journal of clinical psychology.* 1986;42(3):488-492.
5. Trockel M, Bohman B, Lesure E, et al. A Brief Instrument to Assess Both Burnout and Professional Fulfillment in Physicians: Reliability and Validity, Including Correlation with Self-Reported Medical Errors, in a Sample of Resident and Practicing Physicians. *Acad Psychiatry.* 2018;42(1):11-24.

© 2024 Sinsky CA et al. *JAMA Network Open.*
