# Supplementary material for: Vacation Days Taken, Work During Vacation, and Burnout Among US Physicians
Source: JAMA Netw Open. 2024 Jan 12;7(1):e2351635. doi: 10.1001/jamanetworkopen.2023.51635 (PMC10787314; doi:10.1001/jamanetworkopen.2023.51635)
Supplement: Supplement 2. — Data Sharing Statement [file jamanetwopen-e2351635-s002.pdf]

Data Sharing Statement

Sinsky. Vacation Days Taken, Work During Vacation, and Burnout Among US Physicians.
JAMA Netw Open. Published January 12, 2024. doi:10.1001/jamanetworkopen.2023.51635

Data
Data available: No
